# Renal Pelvis Urine Sampling for Microbiology in Patients Undergoing Ureteric Stent Insertion for Infected Obstructed Kidneys: A Departmental Practice Review for Compliance With Standard Care

**DOI:** 10.7759/cureus.100167

**Published:** 2025-12-27

**Authors:** Muhammad Arshad Irshad Khalil, Rory Brennan, Hazel E Smith

**Affiliations:** 1 Urology, National Health Service (NHS) Education for Scotland, Edinburgh, GBR; 2 Urology, Western General Hospital, Edinburgh, GBR

**Keywords:** infected obstructed kidney, percutaneous nephrostomy, renal pelvic urine, ureteric stent insertion, urosepsis

## Abstract

Introduction

Ureteric stent insertion allows the collection of renal pelvic urine (RPU) for microbiology, aiding targeted antibiotic use in infected obstructed kidneys (IOK). This retrospective audit reviewed compliance with European Association of Urology standards for RPU sampling, assessed whether urine dipstick tests and bladder urine samples (BUS) were obtained at initial evaluation, and compared microbiology results from BUS, RPU, and blood samples.

Methods

This retrospective audit reviewed patients with sepsis from suspected IOK who received emergency ureteric stents between October 1, 2024 and February 28, 2025. Microbiological outcomes were evaluated for concordance, and kappa analysis was used to measure agreement.

Results

Among the 51 patients undergoing ureteric stent insertion for IOK, fever was the main indication and obstructing ureteric stones were the most common cause of sepsis. Three quarters of patients lacked a dipstick urine test, and one-fifth had no BUS sent for microbiology at presentation. RPU sampling was not done in 41% of cases during stent insertion. Concordance rates for microbiology results were 63% (RPU vs BUS), 67% (RPU vs blood), and 65% (BUS vs blood). Kappa analysis found fair agreement between RPU and blood, poor agreement between RPU and BUS, and the lowest agreement between BUS and blood microbiology results.

Conclusion

The audit highlights a need to improve compliance with obtaining RPU samples for microbiology, which is vital for managing IOK-driven sepsis. Conducting larger audits in this area can offer more insights into microbial growth and antimicrobial sensitivity patterns.

## Introduction

Urosepsis accounts for approximately 25% of adult sepsis cases requiring hospital admission [1]. Septic shock arising from urosepsis is associated with a significant mortality rate, with 20%-40% of affected patients dying during hospitalization [2]. Infected obstructed kidney (IOK), resulting from various underlying causes, represents a leading contributor to cases of urosepsis [3].

Ureteric stent insertion is a common emergency procedure for IOK in urology, aiming to control sepsis through renal decompression and serving as an alternative to percutaneous nephrostomy (PCN). While debate exists over which method is preferable [4,5], stenting is generally chosen if the patient can undergo general anaesthesia, as it does not require a radiologist, a facility that may not be readily available. The procedure allows aspiration of renal pelvic urine (RPU) for microbiological testing, aiding in the identification of the causative organism and selection of effective antibiotics. The European Association of Urology (EAU) strongly recommends obtaining this sample for microbiology [6].

The main goal of this audit is to assess departmental compliance with current standards of care. In addition, it evaluates whether patients with possible IOK receive proper initial investigations, such as urine dipstick analysis and bladder urine sample (BUS) testing. The audit also examines the agreement between RPU, BUS, and blood microbiology results to determine the most effective sample for identifying sepsis-causing organisms. By highlighting compliance gaps, this audit seeks to improve clinical practice and patient care quality.

## Materials and methods

We retrospectively reviewed records of all patients who underwent emergency ureteric stent insertion for radiologically obstructed kidneys at Western General Hospital Edinburgh from October 1, 2024 to February 28, 2025. Included patients had suspected infected obstructed kidneys with clinical or biochemical evidence of infection, defined by a temperature ≥38.5°C and/or elevated inflammatory markers, including C-reactive protein ≥20 mg/L or white cell count ≥11×10⁹/L. Patients treated solely for pain control or renal impairment were excluded.

Variables were categorized and presented using graphs, charts and tables. Figures were used to illustrate concordance in microbiological results, defined as both samples growing the same bacteria or neither showing growth, and discordance, that is, with differing results. Those showing mixed growths were excluded. Cohen's kappa analysis was used to measure agreement among BUS, RPU, and blood sample microbiology, with the kappa statistic influenced by the prevalence and distribution of positive and negative culture results across sample types. Analyses were conducted in R Studio 4.3.3 (Posit Software, Boston, MA).

## Results

**Figure 1:**
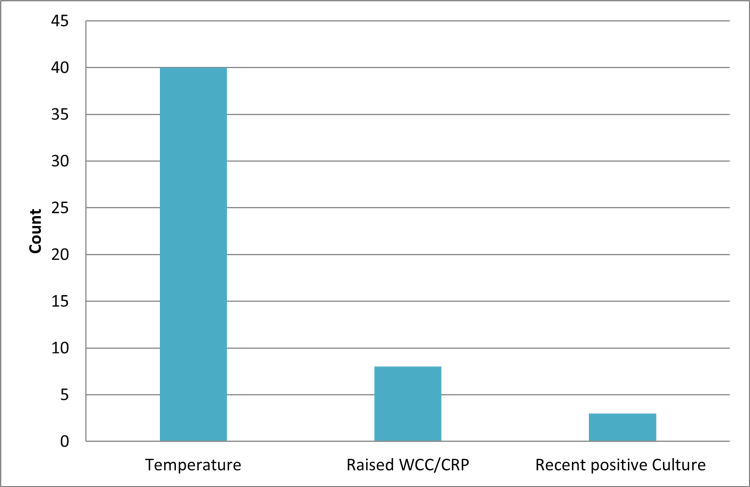
Indications for ureteric stent insertion CRP: C-reactive protein; WCC: white cell count.

**Figure 2:**
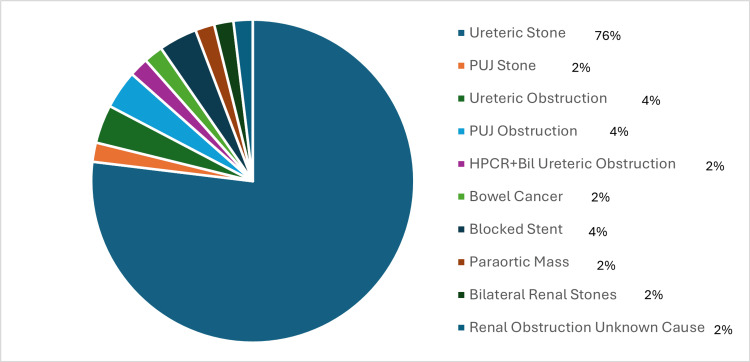
Aetiologies for renal obstruction HPCR: High-pressure chronic retention; PUJ: pelviureteric junction.

Fifty-one patients met the inclusion criteria for ureteric stent insertion. The mean C-reactive protein (CRP) was 161.42 (SD=111.78) and mean white cell count (WCC) was 15.12 (SD=7.32). Figures 1, 2 show the indications and causes for stent insertion and IOK, respectively.

At triage, 75% of patients lacked dipstick urine analysis and 20% did not have BUS microbiology testing. Over half did not have blood samples sent for microbiology. During emergency ureteric stent insertion, RPU sampling was omitted in 41% of cases. Among the remaining 59%, samples were collected in 55% and attempted but not completed in 4%. Table 1 details the sampling and microbiology results.

**Table 1:** Distribution of sampling and microbiology results from BUS, RPU and blood BUS: Blood urine samples; RPU: renal pelvic urine

Specimen	Not Sampled, n (%)	Sampled, n (%)	Microbiology Result		
			Growth, n (%)	No Growth, n (%)	Mixed Growth, n (%)
BUS	10 (19.6)	41(80.3)	14 (34.1)	25 (60.9)	2 (4.8)
RPU	23 (45.1)	28 (54.9)	8 (28.5)	19 (67.8)	1 (3.5)
Blood	28 (54.9)	23 (45.1)	5 (21.7)	18 (78.3)	0

Analysis of concordance

**Figure 3:**
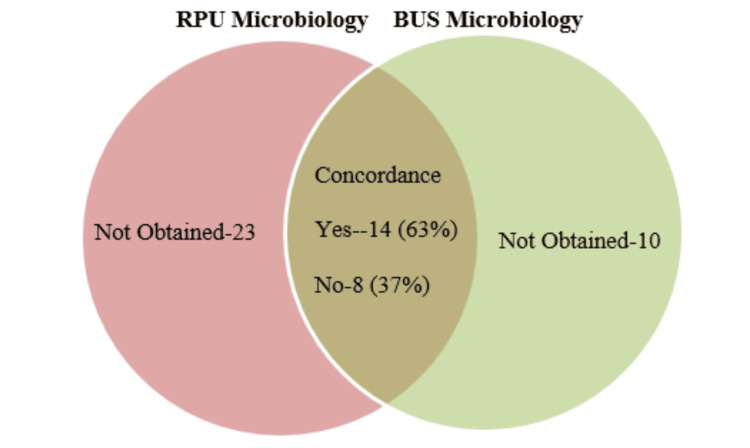
Comparison of RPU and BUS microbiology outcomes RPU: Renal pelvic urine; BUS: blood urine sample

**Figure 4:**
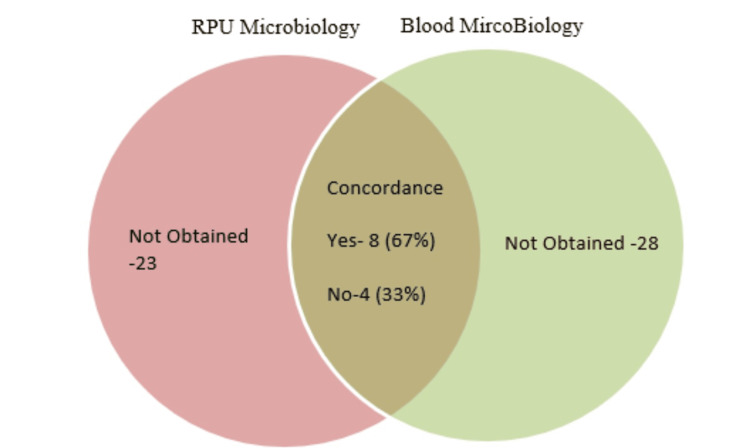
Comparison of RPU and blood microbiology outcomes RPU: Renal pelvic urine

**Figure 5:**
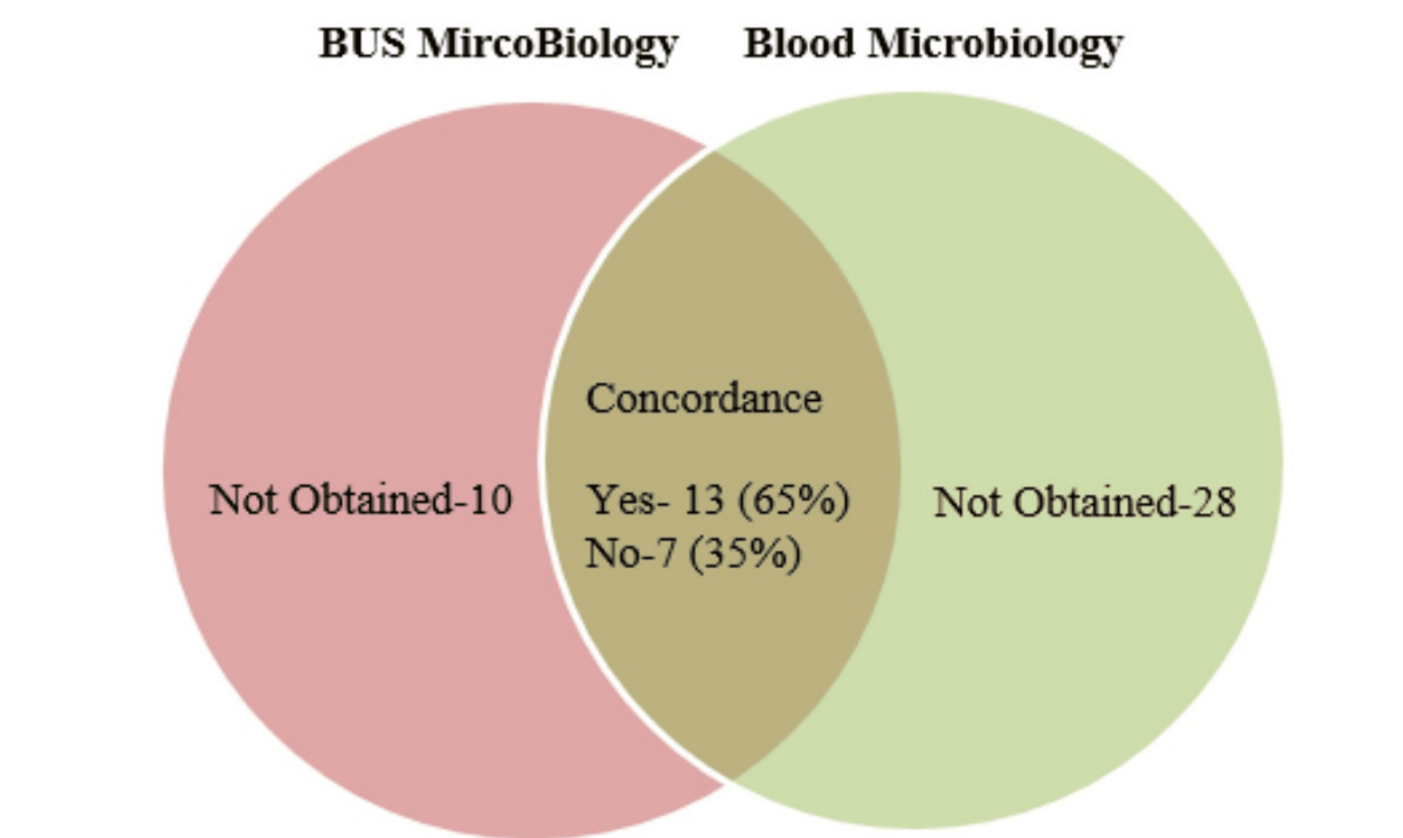
Comparison of BUS and blood microbiology outcomes BUS: blood urine sample.

Figures 3-5 show that concordance analyses of microbiology results between RPU, BUS, and blood samples are consistent across categories. However, a key limitation is insufficient sampling in all groups.

Kappa analysis results

On kappa analysis, the kappa statistic demonstrated fair agreement between RPU and blood microbiology (0.25; 95% CI: -0.03 to 0.53), and poor agreement between RPU and BUS microbiology (0.17; 95% CI: -0.01 to 0.35). Notably, the statistic was negative between BUS and blood microbiology (-0.21), reflecting even lower concordance.

The results from the concordance and kappa analyses indicate that microbiological growths identified in RPU samples exhibit greater alignment with those found in blood samples. In contrast, the observed concordance between bladder urine and blood cultures was driven entirely by concordant no-growth results, as no identical bacterial organisms were isolated from both samples.

## Discussion

Infection, complicated by urinary tract obstruction (IOK), is a leading cause of urosepsis [7], with urolithiasis being the most common underlying factor based on our audit and other studies [8]. IOK requires immediate urological intervention. Although both ureteric stent placement and PCN are valid drainage methods, no clear preference exists [9,10]. Typically, ureteric stents are favoured, as they can be inserted independently by urologists, whereas PCN insertion is reliant on radiologists who may be less accessible, potentially delaying sepsis management and affecting outcomes.

Intraoperative RPU sampling has been well studied in the context of percutaneous nephrolithotomy [11], with evidence suggesting that RPU samples better identify sepsis-causing organisms [12] and guide antibiotic selection. The EAU guidelines thus strongly recommend obtaining these samples [6]. The same approach should apply to patients with potentially infected obstructed kidneys. However, evidence remains limited, and this audit aims to encourage further research.

Urine dipstick analysis is a valuable point-of-care test in sepsis management, helping identify urinary tract infections quickly and guiding timely antibiotic treatment. It offers strong positive and negative predictive values for diagnosing urinary tract infections (UTIs) [13] and is almost universally accessible. However, this audit showed that it was significantly underused during initial patient triage, occurring in only 25% of cases. Additionally, 20% of cases did not have BUS samples sent for microbiological testing. Missing these results can hinder effective sepsis management.

In this audit, we found a 37% discordance between BUS and RPU microbiology results. BUS microbiology was not performed in 20% of patients during initial triage, yet the discordance rate aligns with previous findings. Mirzazadeh et al. reported a 31% mismatch [12], and Yoshida et al. observed similar patterns in ureteroscopy patients [14].

The concordance between bacterial growth in RPU and blood samples is 67%, with kappa analysis indicating fair agreement. Within the limitations of being a single-centre audit and incomplete sampling, RPU samples appeared more closely aligned with blood culture findings than bladder urine samples. This finding supports observation in other studies [12,15,16] reporting higher concordance (up to 95%), and thus advocates utilization RPU microbiology for controlling IOK-driven urosepsis.

Being a single-centre audit, this study is limited by a relatively small sample size, which restricts the depth of analysis regarding microbial growth and antibiotic sensitivity patterns. Nevertheless, the findings are sufficient to underscore non-compliance with current standards and can serve as a foundation for future research, ideally involving multiple centres.

## Conclusions

Compliance with the standard of obtaining RPU samples for microbiology, during ureteric stent insertion in patients with sepsis from IOK is suboptimal. Adding this step to theatre checklists and trainee assessments may improve adherence. RPU microbiological results may provide valuable information in managing IOK-driven sepsis. Larger, preferably multi-centre audits in this area are needed to assess microbial growth trends and antimicrobial sensitivity patterns.
